# Supplementary material for: ATF6 Activation Reduces Amyloidogenic Transthyretin Secretion through Increased Interactions with Endoplasmic Reticulum Proteostasis Factors
Source: Cells. 2022 May 17;11(10):1661. doi: 10.3390/cells11101661 (PMC9139617; doi:10.3390/cells11101661)
Supplement: Supplementary file 1 [file cells-11-01661-s001.zip › cells-1704146-supplementary.pdf]

cells MDPI

Supplement to:

ATF6 Activation Reduces Amyloidogenic Transthyretin Secretion Through Increased Interactions with Endoplasmic Reticulum Proteostasis Factors

Jaleh S. Mesgarzadeh[1,*], Isabelle C. Romine[1,*], Ethan M. Smith-Cohen[1], Julia M.D. Grandjean[1], Jeffery W. Kelly[2,3] Joseph C. Genereux[2,4], and R. Luke Wiseman[1,5]

[1]Department of Molecular Medicine, The Scripps Research Institute, La Jolla, CA

[2]Department of Chemistry, The Scripps Research Institute, La Jolla, CA

[3]The Skaggs Institute for Chemical Biology, The Scripps Research Institute, La Jolla, CA

[4]Department of Chemistry, University of California, Riverside, Riverside, CA

*These authors contributed equally

[5]To whom correspondences should be addressed:

R. Luke Wiseman

Department of Molecular Medicine

The Scripps Research Institute

MB110

La Jolla, CA 92037

Email: wiseman@scripps.edu

Phone: (858) 784-8820

Running title: ATF6-dependent retention of TTR

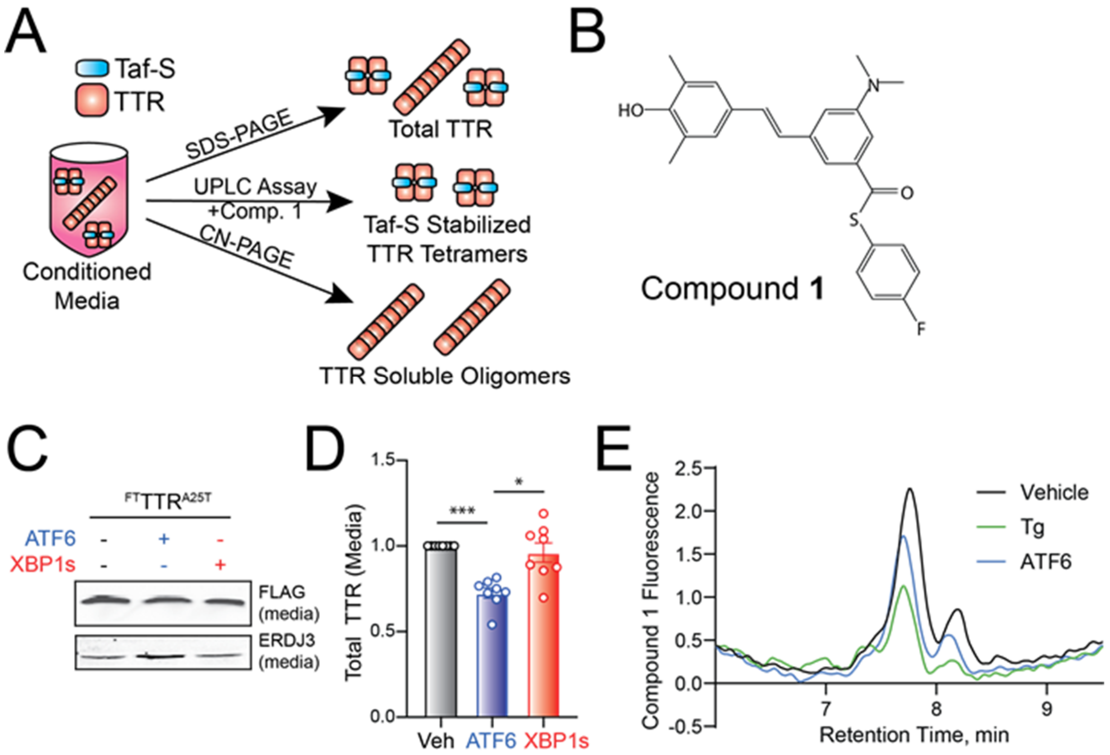

Figure S1. (Supplement to Figure 1). A. The three assays used to monitor total, aggregate, and tetrameric TTR. This 661 figure is adapted from Ref 21. B. Structure of compound 1. Upon binding to the native TTR tetramer, compound 1 covalently modifies TTR at Lys15, allowing fluorescence. Please see Refs 21 and 22 for additional details. C,D. Representative immunoblot and quantification of FTTTRA25T in conditioned media prepared on HEK293DAX cells following TMP-dependent DHFR.ATF6 activation (ATF6) or dox-dependent XBP1s activation (XBP1s). Conditioned media was prepared for 18 h. ERdj3 is an ATF6-regulated secreted chaperone that is shown as a control. Error bars show SEM for n=8 independent replicates. *$p<0.05$, ***$p<0.005$ for an RM one-way ANOVA relative to Veh-treated cells. E. Representative anion exchange chromatogram for FTTTRA25T tetramers in conditioned media prepared on HEK293DAX cells following treatment with Tg (500 nM) of TMP-dependent DHFR.ATF6 activation (ATF6).

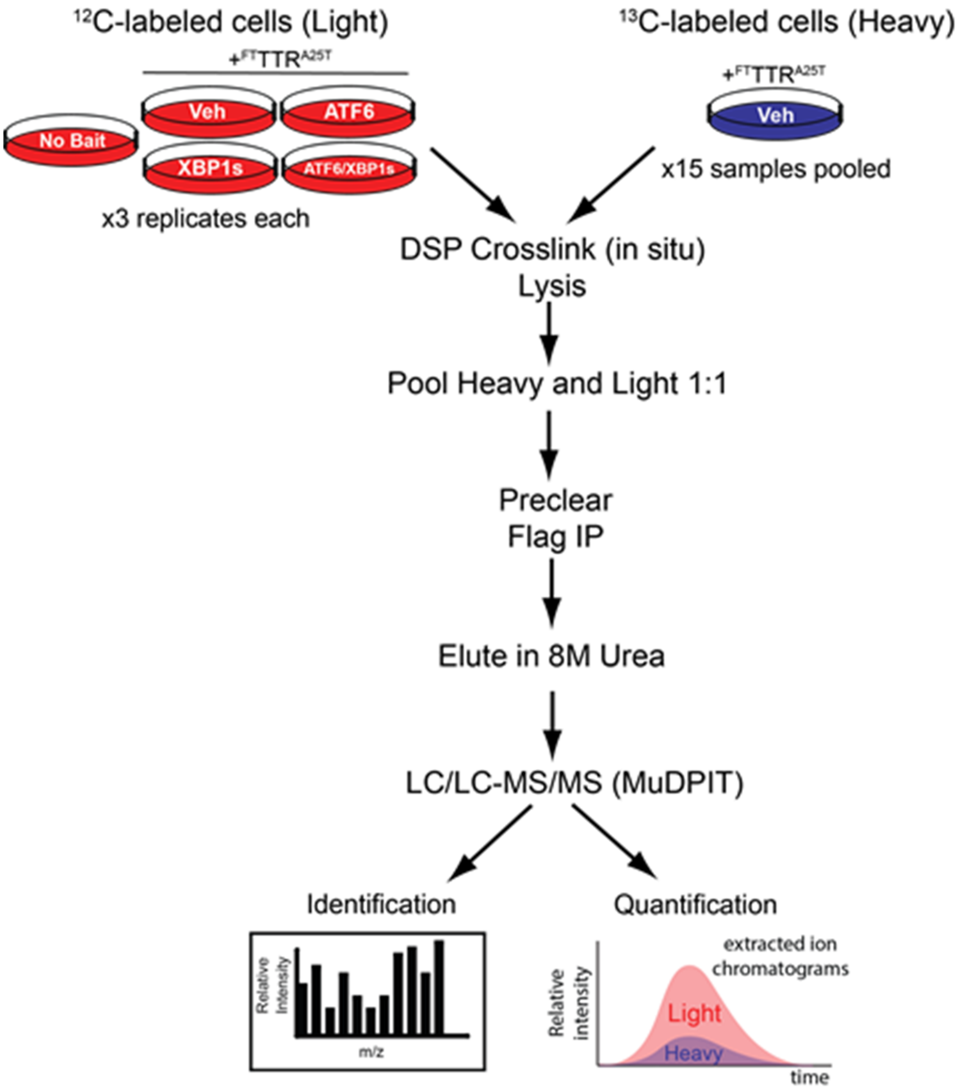

Figure S2. (Supplement to Figure 2). Transfected and treated cells were cultured in either Light (^{12}C-labeled) or Heavy (^{13}C-labeled) media. After labeling, cells were collected and crosslinked with DSP in situ then subsequently lysed. Cell lysates were pooled 1:1 (one Light lysate paired with equivalent Heavy lysate amount) and then precleared with Sepharose 4B beads. After clearing, the supernatants were placed onto M1 anti-flag beads for IP. The M1 beads were eluted using 8M urea and submitted to LC/LC-MS/MS MuDPIT analysis to identify and quantify the ratio of Light versus Heavy labeled proteins.

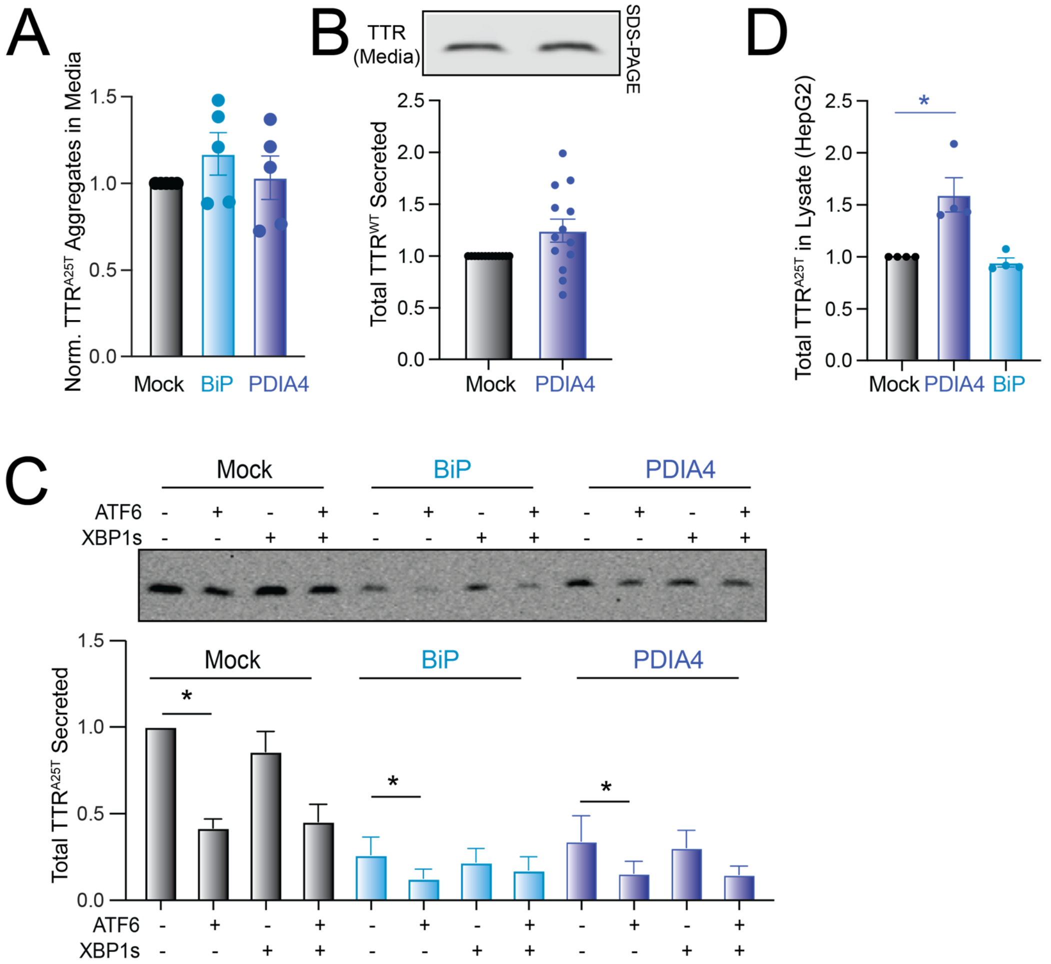

Figure S3. (Supplement to Figure 3). (A). Normalized FTTTRA25T aggregates in conditioned media prepared on HEK293T cells overexpressing Mock, BiP, or PDIA4. Media was conditioned for 18 h. Normalized aggregates was calculated as in Fig. 1E. Error bars show SEM for n=5 independent replicates. (B). Representative SDS-PAGE immunoblot and quantification of FTTTRWT in conditioned media prepared on HEK293T cells overexpressing Mock or PDIA4. Error bars show SEM for n= 13 replicates. (C). Representative immunoblot and quantification of FTTTRA25T in conditioned media prepared on HEK293DAX cells overexpressing Mock, BiP, or PDIA4 and treated with trimethoprim (TMP; activates ATF6) and/or doxycycline (dox; activates XBP1s), as indicated. Error bars show SEM for n=3 replicates. *indicates $p<0.05$ for a one-tailed paired t-test. (D). Quantification of FTTTRA25T in lysates prepared on HepG2 cells overexpressing Mock, PDIA4, or BiP, as indicated. A representative immunoblot is shown in Fig. 3D. Error bars show SEM for n=4 replicates. *indicates $p<0.05$ for one-way ANOVA.

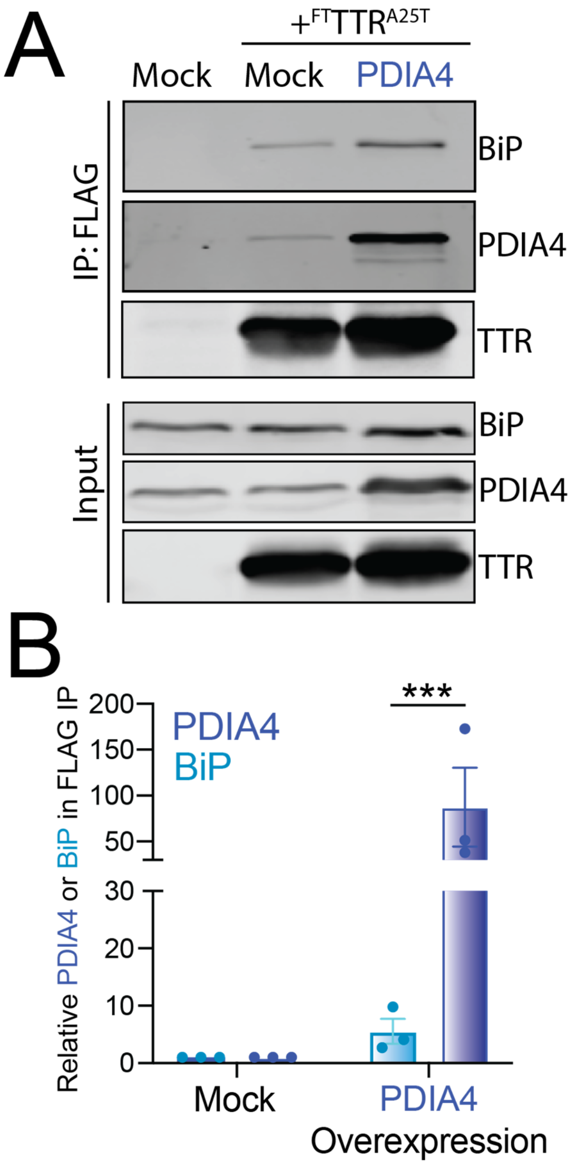

Figure S4. (Supplement to Figure 4). PDIA4 or BiP overexpression increases ER retention of destabilized FTTTRA25T.(A,B). Representative immunoblot and quantification of PDIA4 and BiP in FLAG immunopurifications from HEK293T cells transiently expressing FTTTRA25T and transfected with mock or PDIA4. The relative recovery of PDIA4 or BiP was normalized to the relative recovery of FTTTRA25T from each condition. Error bars show SEM for n=3 independent experiments. ***p<0.005 for a paired t-test.

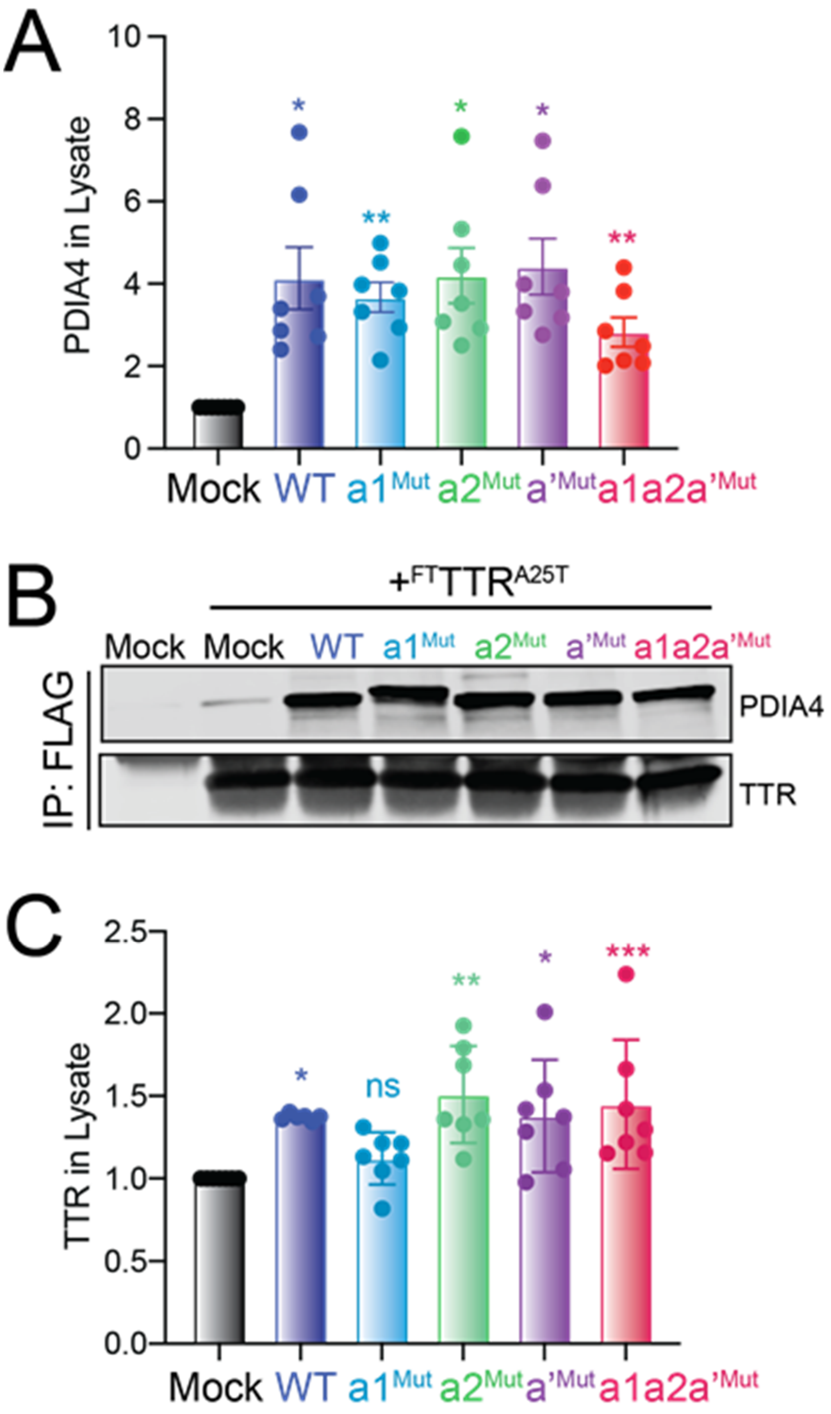

Figure S5. (Supplement to Figure 5). (A). Quantification of PDIA4 in lysates prepared on HEK293T cells overexpressing the indicated PDIA4 variant. A representative immunoblot is shown in Fig. 6B. Error bars how SEM for n=7 replicates. (B). Immunoblot of FLAG immunopurifications from HEK293T cells overexpressing FTTTRA25T and the indicated PDIA4 variant. A representative immunoblot of inputs for this experiment is shown in Fig. 6B. (C). Quantification of TTR in lysates prepared on HEK293T cells overexpressing the indicated PDIA4 variant. A representative immunoblot is shown in Fig. 6B. Error bars show SEM for n=7 replicates. *$p<0.05$, **$p<0.01$, ***$p<0.005$ for an RM one-way ANOVA relative to Mock-transfected cells.

Table S1. (Supplement Table to Figure 2). 'True' interactor proteins shown to have a >2-fold enrichment in FTTTRA25TIPs, relative to mock IPs, measured by SILAC-MuDPIT proteomics.

Protein	Enrichment	ER Localized?
TTR^{A25T}	65.67	N/A
DNAJB11	28.8	YES
BiP(GRP78)	24.11	YES
SDFL2	23.2	YES
GRP94	21.2	YES
TFR1	18.59	NO
ROA2	16.96	NO
UGGG1	15.85	YES
H4	15.24	NO
PDIA6	15.15	YES
GT251	12.97	YES
DJC10	12.83	YES
TXND5	12.06	YES
PDIA1	11.87	YES
SAR1A	11.5	YES
CALX	10.36	YES
PDIA3	10.03	YES
GLU2B	9.53	YES
SERPH	9.42	YES
PSMD3	8.67	NO
ERP29	8.1	YES
PPIB	7.4	YES
GANAB	7.13	YES
FKBP4	6.86	NO
PDIA4	6.84	YES
PRDX4	6.65	YES
CLUS	5.57	NO
ERP44	5.43	YES
CALR	5.33	YES
CS010	5.07	NO
ERO1A	4.94	YES
SC22B	4.8	YES
RCN2	4.38	YES
RPN2	4.31	YES
OST48	4.2	YES
ATPB	4.14	NO
CNPY2	4.11	YES
ESYT1	**4.06**	**YES**
RPN1	3.58	YES
NPC2	3.33	YES
LDHA	3.32	NO
ATPA	3.3	NO
LDHB	3.08	NO
NOMO2	3.02	YES
NOMO1	3.02	YES
NOMO3	3.02	YES
KCRB	2.93	NO

RANG	2.93	NO
DNS2A	2.91	NO
TERA	2.81	YES
ACTG	2.57	NO
ACTB	2.57	NO
RAB1B	2.47	NO
RAB1C	2.47	NO
VDAC2	2.32	NO
RAB1A	2.32	NO
EF1G	2.26	NO
C1QBP	2.25	NO
PCBP1	2.21	NO
MPCP	2.15	NO
PSB5	2.15	NO
KPYM	2.11	NO
HYOU1	2.11	YES
RAB7A	2.11	NO
K6PP	2.06	NO

Table S2. (Supplement Table to Figure 2). Log_2 fold-change, -log FDR, and adjusted q-values of 'true $^{FT}TTR^{A25T}$ interactors' detected in lysates prepared from $HEK293^{DAX}$ cells following treatments with trimethoprim (TMP; activates ATF6) or doxycycline (dox; activates XBP1s), as compared to vehicle-treated cells. Measured by SILAC-MuDPIT proteomics. Note that only 'true' interactors identified under these conditions are shown.

.	TMP			Dox		
**Protein**	**Log2(fold-change)**	**-Log(FDR)**	**Adj. q-value**	**Log2(fold-change)**	**-Log(FDR)**	**Adj. q-value**
BiP(GRP78)	1.13504691	2.22464946	0.00596143	0.1147528	0.34548025	0.45135655
GRP94	1.43849948	2.27859202	0.00526512	0.14659066	0.21387119	0.61112325
PDIA4	1.50950698	2.74295886	0.00180735	0.62795032	0.69990068	0.19957187
TERA	1.29681058	0.59741915	0.27442559	0.62263588	0.41444217	0.38508609
RPN1	0.57938326	0.53264346	0.29333004	-0.1091652	0.0365344	0.91931766
HYOU1	0.93070673	1.71395977	0.01932147	0.49297069	0.60940567	0.24580705
SDF2L	0.93727183	1.89203376	0.01282231	0.67198103	0.60940567	0.24580705
SC22B	0.50430601	0.39866829	0.39932979	0.1577241	0.36196355	0.43454669
PDIA1	0.50673323	0.8051127	0.15663445	0.3103115	0.69990068	0.19957187
PDIA6	0.23754837	0.56715766	0.31480899	0.08622505	0.34548025	0.45135655
CALR	0.75451674	1.45283846	0.0352502	0.33117101	0.36196355	0.43454669
DNAJB11	0.9055241	0.85019043	0.14119183	0.59127896	1.34698829	0.0449792
PDIA3	0.1899624	0.56715766	0.27092079	0.06171541	0.21387119	0.61112325
TXND5	-0.1847943	0.24140602	0.57357998	0.69695298	0.60940567	0.24580705
GLU2B	0.04783636	0.06474213	0.86150513	-0.3824585	0.56320469	0.27339799
RAB1A	0.93731199	0.67634386	0.21069593	1.04732261	0.36196355	0.43454669
SERPH	-0.2036718	0.36389023	0.43262316	-0.0911674	9.7621E-05	0.99977524
PRDX4	-0.0641237	0.13344081	0.73546022	-0.194222	0.12340934	0.75264583
CLUS	1.15749124	0.30865777	0.50686318	0.18992528	0.69990068	0.19957187
ERP29	-0.6586387	0.8051127	0.17548768	-0.3821885	0.36196355	0.43454669
ERP44	-0.1043777	0.14565963	0.71505651	-0.1279065	9.7621E-05	0.99977524
CALX	0.47888689	0.3048391	0.49563378	-0.0365977	0.06027923	0.87040379
CS010	-0.2694868	0.29719637	0.50641322	-0.0501039	0.07278304	0.84570123

UGGG1	-0.159551	0.24140602	0.57531923	-0.0977695	0.00997183	0.97730062
PPIB	-0.2294148	0.56715766	0.31568957	0.03112861	0.35231506	0.44430882
ERO1A	-0.4060671	0.53264346	0.30063935	-0.3454872	0.36196355	0.43454669
RCN2	1.61482062	0.29719637	0.50443316	-0.2414799	0.2260069	0.59428272
CNPY2	-0.3180001	0.30865777	0.4958591	-0.2375996	0.0365344	0.91931766
RAB1B	1.01759711	0.39866829	0.41443987	0.63031831	0.69990068	0.19957187
GT251	-0.7820825	0.59741915	0.25268581	-0.6098503	0.60940567	0.24580705
DNAJC10	0.29626762	0.56715766	0.27430042	0.34737888	0.34277206	0.45417993
NOMO2	1.81495724	0.56715766	0.31643915	0.17095622	0.33067178	0.4670122
NOMO1	1.81495724	0.56715766	0.31643915	0.17095622	0.33067178	0.4670122
NOMO3	**1.81495724**	**0.56715766**	**0.31643915**	**0.17095622**	**0.33067178**	**0.4670122**
GANAB	0.42571996	0.30865777	0.49129487	-0.0823035	0.0365344	0.91931766
NPC2	0.48701591	0.39866829	0.40230175			
RPN2	-0.2394572	0.24140602	0.57773744			
